# On-Chip DNA Methylation Analysis Using Osmium Complexation

**DOI:** 10.4061/2011/480570

**Published:** 2011-05-15

**Authors:** Kaori Sugizaki, Tadashi Umemoto, Akimitsu Okamoto

**Affiliations:** ^1^Nucleic Acid Chemistry Laboratory, RIKEN Advanced Science Institute, 2-1 Hirosawa, Wako, Saitama 351-0198, Japan; ^2^PRESTO, Japan Science and Technology Agency, 4-1-8 Honcho, Kawaguchi, Saitama 332-0012, Japan

## Abstract

The development of a reaction for detecting the presence/absence of one methyl group in a long DNA strand is a chemically and biologically challenging research subject. A newly designed chemical assay on a chip for the typing of DNA methylation has been developed. A methylation-detection probe fixed at the bottom of microwells was crosslinked with methylated DNA mediated by osmium complexation and contributes to selective amplification of methylated DNA.

## 1. Introduction

Gene expression is well regulated by the epigenetic modification of DNA and histone tails independent of their primary sequences. In particular, cytosine methylation, in which the C5 position of the cytosine base is methylated enzymatically, plays a crucial role in the regulation of chromatin stability, gene regulation, parental imprinting, and X-chromosome inactivation in females [1–4]. Therefore, detection of cytosine methylation is very important, and much effort has gone into developing a simple reaction for 5-methylcytosine (^m^C) detection.

For the evaluation of the methylation status of genes, several conventional methods have so far been used, such as a cleavage assay with methylation-insensitive restriction enzymes [5–7], hydrolysis and sequencing with a bisulfite salt [8–10], and immunofluorescence with anti-5-methylcytosine antibody [11, 12]. Although the conventional methods have many merits, there are many disadvantages, and methylation detection assays must be further improved through another approach. The existence of a more rapid and selective chemical reaction capable of distinguishing between methylcytosine and unmethylated cytosine on a chip has promise as a good method for efficiently analyzing the status of cytosine methylation at a specific site in a gene.

**Figure 1:**
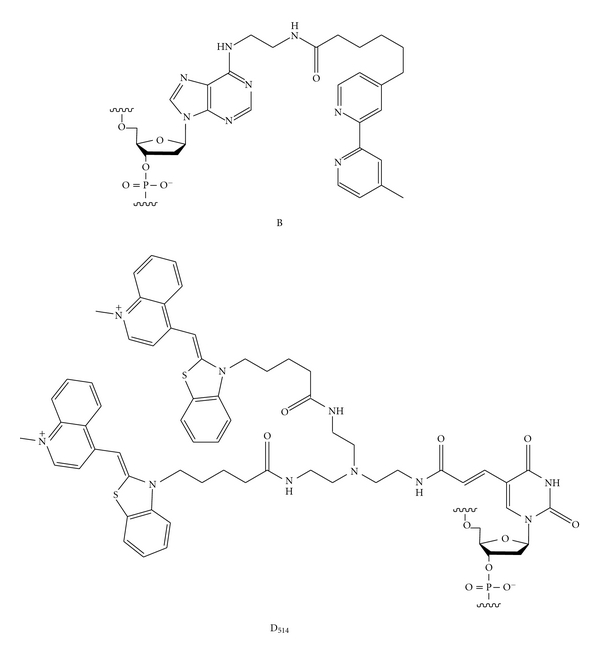
Structures of the “B” nucleotide of the ICON probe for 5-methylcytosine selective crosslink formation and the “D_514_” nucleotide of the Exciton primer for real-time PCR monitoring.

The sequence-selective DNA methylation-detection probe, ICON (interstrand complexes formed by osmium and nucleic acids), may be effective for the development of an on-chip analysis of DNA methylation [13–15]. In the presence of osmium oxidants and a bipyridine ligand, 5-methylcytosine forms a stable osmium-centered complex, in contrast to unmethylated cytosine (Figure 1) [16–19]. ICON probes form a crosslink with a specific 5-methylcytosine in the probe-hybridizing DNA mediated by osmium-centered complex formation. This function will be effective for the capture of methylated DNA on a chip for sequence-selective methylation analysis.

In this paper, development of an on-chip analyzing method for typing of DNA methylation at a specific cytosine is reported. ICON probes fixed to the bottom of microwells assisted the on-chip detection of the methylation status of a specific cytosine in the target DNA.

## 2. Materials and Methods

### 2.1. Synthesis of an ICON Probe

Artificial DNA was synthesized by the conventional phosphoramidite method using an Applied Biosystems 392 DNA/RNA synthesizer or an NTS H-6 DNA/RNA synthesizer. The phosphoramidite form of bipyridine-modified adenine (B) was prepared according to the synthetic protocol described in a previous paper [13]. The 5′-amino end was attached using the phosphoramidite of 5′-aminomodifier C12 (Glen Research (http://www.glenresearch.com/index.php)). Synthesized DNA was purified by reverse phase HPLC on a 5-ODS-H column (10 mm × 150 mm, elution with a solvent mixture of 0.1 M triethylammonium acetate (TEAA), pH 7.0, linear gradient over 30 min from 5% to 20% acetonitrile at a flow rate of 3.0 mL/min). The DNA strand was characterized by MALDI-TOF MS. 5′-NH_2_-(CH_2_)_12_-CCCCCCCCCCCACAACCTCCBTCATGTGCTGAA-3′ ([M + H]^+^, calcd. C_343_H_452_N_118_O_198_P_33_, 10418.0, found 10416.0).

### 2.2. Preparation of Chips

 A 100 *μ*L solution of synthetic DNA (100 nM) in TE buffer (pH 7.0) or deionized water in the presence of 10 mM 1-ethyl-3-(3-dimethylaminopropyl)carbodiimide, and 10 mM 1-methylimidazole was put into each well of NucleoLink strips (Nalge Nunc (http://www.nalgenunc.com/)). After incubation at 50°C for 5 h, the reaction mixture was removed from the well, and the well was rinsed three times with a solution of 100 mM Tris-HCl (pH 7.5), 150 mM sodium chloride, and 0.1% Tween20, and then three times with deionized water.

### 2.3. Osmium Treatment and DNA Amplification

The target DNA sequence p53(N^1^–N^2^) was 5′-TGT GCA GCT GTG G GTT GAT T CGA CAC CCC C GCC CGG CAC C CGC GTC CGC G CCA TGG CCA T CTA CAA GCA G TCA CAG CAC A TGA N^1^GG AGG T TGT GAG G N^2^G C TGC CCC CAC C-3′ (N^1^, N^2^ = C or ^m^C). A 25 *μ*L solution of DNA (8 nM) in 50 mM Tris-HCl buffer (pH 7.7), 0.5 mM EDTA, and 1 M sodium chloride was added to each probe-attached well at 0°C. The reaction mixture was incubated at 0°C for 5 min, and then the solution was removed from the wells. A 25 *μ*L solution of 5 mM potassium osmate(VI) and 100 mM potassium hexacyanoferrate(III) in 50 mM Tris-HCl buffer (pH 7.7), 0.5 mM EDTA, and 1 M sodium chloride was incubated at 0°C for 5 min or at 25°C for 10 min. The wells were rinsed seven times with 0.4 M sodium hydroxide, 0.25% Tween20 (130 *μ*L/well). After further rinsing with deionized water twice, the wells were coated with 10 mg/mL BSA in 100 mM Tris-HCl (pH 7.5), 150 mM sodium chloride, and 0.1% Tween20. The process of PCR amplification was performed in a reaction solution of 1 U *TaKaRa Ex Taq *HS, 10× buffer, 2.5 mM dNTP mix, and 1 *μ*M primer mix (Forward, 5′-AGCTGD_514_GGGTTGATTC-3′ for the Exciton primer method, 5′-TGTGCAGCTGTGGGTTGATTC-3′ for the SYBR Green I method; reverse, 5′-ACTGCTTGTAGATGGCCATG-3′; D_514_ in an Exciton primer is a hybridization-sensitive fluorescent nucleotide (Figure 1)). In the case of using the stain method with SYBR Green I fluorescence for monitoring the amplification, SYBR Green I dye was also added to the reaction mixture in advance. Amplifications were performed in microwells as follows: after heating at 95°C for 60 s, 35 cycles of denaturation at 95°C for 5 s, annealing with fluorescence monitoring at 52°C for 20 s, and extension at 72°C for 20 s on the Corbett Rotor-Gene, the amplification process was monitored by the fluorescence of D_514_ or SYBR Green I through an SYBR Green I (470 nm/510 nm) filter.

## 3. Results and Discussion

### 3.1. Preparation of Chips and Osmium Complexation

**Figure 2:**
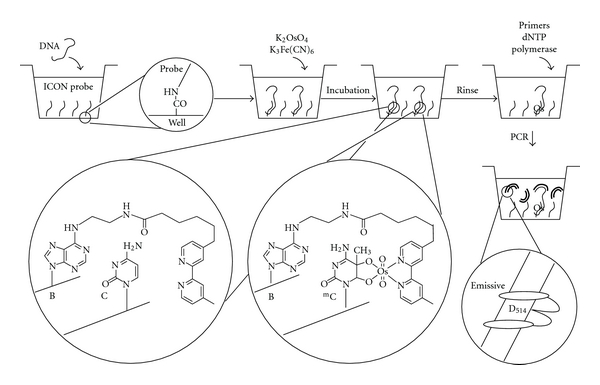
Schematic illustration of on-chip analysis of methylated DNA.

For the on-chip study, we adopted a NucleoLink strip, because it is a microwell strip in which the amino-modified ICON probe can be attached to well bottoms with covalent bonds. The target DNA was a fragment of the human p53 gene exon 5 including mutation hotspots at CpG dinucleotides [20]. The sequence of the ICON probe was designed to form a crosslink with one of the methylated cytosines in the target DNA. The ICON probe was modified with an amino end for attaching to the plate and an alkyl linker and a poly C sequence for introducing enough distance between the probe and the plate. This ICON probe was synthesized by the conventional phosphoramidite method by using a DNA autosynthesizer. The phosphoramidite form of bipyridine-modified adenine (B) was prepared according to the synthetic protocol described in a previous paper [13]. Synthetic DNA was fixed to the bottom of each well of NucleoLink strips in the presence of 1-ethyl-3-(3-dimethylaminopropyl)carbodiimide and 1-methylimidazole (Figure 2). The wells were rinsed and used for the next reaction.

Oxidative osmium complexation using ICON probes is a rapid and mild reaction for detection of methylated DNA, and it is not accompanied with nonspecific strand damage, in contrast to conventional bisulfite methods. Therefore, this reaction would be effective for on-chip analysis of DNA methylation. The target DNA was put into the wells and hybridized with the ICON probes fixed in the wells. The DNA samples in the wells were incubated at 0°C for 5 min then at 25°C for 5 min in the presence of 5 mM potassium osmate(VI) and 100 mM potassium hexacyanoferrate(III) in 50 mM Tris-HCl buffer (pH 7.7), 0.5 mM EDTA, and 1 M sodium chloride. After reaction, the wells were rinsed with 4 M sodium hydroxide and then coated with BSA.

### 3.2. Exciton Primer and Real-Time PCR Monitoring

**Figure 3:**
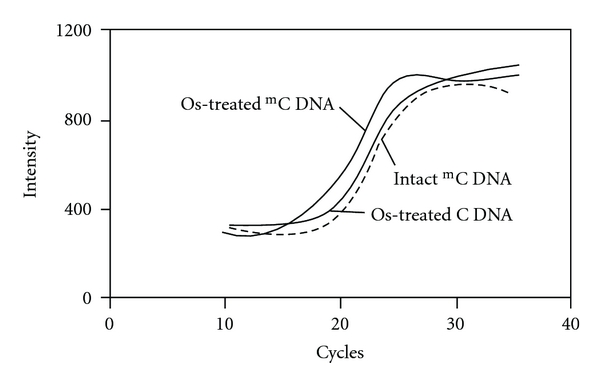
Profile of real-time PCR for p53(^m^C–C) and p53(C–C) with/without osmium oxidation.

The fixed DNA was detected using PCR amplification of a part of the crosslinking DNA strand. The PCR primers were designed for the region without ICON binding. For one of the primers, a hybridization-sensitive fluorescent DNA was used, containing a fluorescent nucleotide D_514_ (Figure 1) [21–24]. This fluorescent DNA shows very weak fluorescence in the unhybridized state, whereas it shows strong fluorescence after hybridization with the complementary nucleic acids. This fluorescence switching is controlled by an intramolecular excitonic interaction between dyes tethered to the DNA. This fluorescent DNA is useful as a PCR primer. This DNA shows weak fluorescence emission, whereas the PCR mixture emits strong fluorescence after PCR amplification. This system has been applied to the detection of single nucleotide polymorphisms in genome DNA samples [25]. We tested this “Exciton primer” for on-chip PCR. The process of PCR amplification was performed in a reaction solution of TaKaRa Ex Taq HS polymerase in the presence of a mixture of dNTP and primer mix. Amplifications were performed in microwells and the change in the fluorescence intensity monitored using a real-time PCR system. In the experiment for p53(^m^C–C) and p53(C–C), the methylation of the target cytosine was determined from the increase in the fluorescence signal associated with the exponential growth of the PCR product. Amplification of the p53(^m^C–C) started first, and then the amplification of p53(C–C) started several cycles later (Figure 3). Washingout of uncrosslinked sample DNA brought about this lag in the starting point of amplification. The amplification curve observed for p53(C–C) almost overlapped that for the osmium-untreated p53(^m^C–C), suggesting that the amplification curve for unmethylated DNA is due to amplification of the DNA nonspecifically adsorbing to the well surface. On-chip capture of methylated DNA by the ICON probe at the methylation site made possible the sequence specific detection of methylation through PCR amplification.

### 3.3. Sequence-Specific Amplification

**Figure 4:**
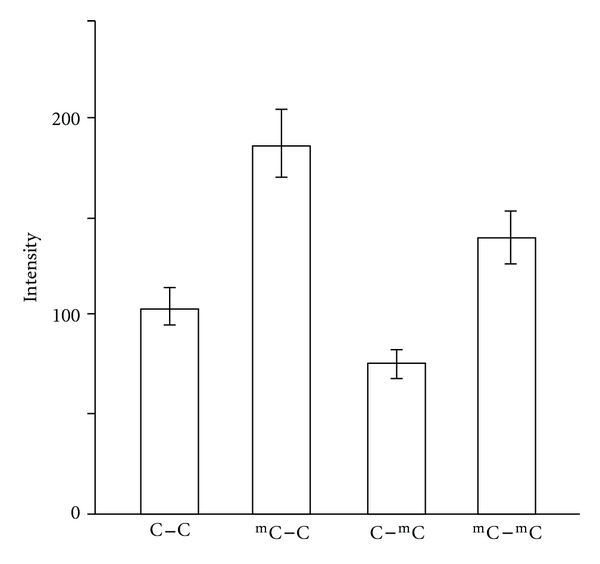
Sequence-specific detection of DNA methylation at N^1^ in p53(N^1^–N^2^) using an ICON probe.

A prototype for ICON-based on-chip methylation analysis makes possible sequence-specific amplification. We prepared four DNA strands with different methylation sites, p53(C–C), p53(^m^C–C), p53(C–^m^C), and p53(^m^C–^m^C). The sample DNA was added to the wells, in which the ICON probe targeting only the ^m^C of the sample DNA 5′ side was fixed. After osmium treatment and BSA coating, the crosslinked DNA was amplified by PCR in the presence of unlabeled primers and SYBR Green I. After 15 cycles of the amplification reaction, the fluorescence intensity of SYBR Green I was quantified on a microplate reader (Figure 4). The wells containing p53(^m^C–C) and p53(^m^C–^m^C) exhibited higher fluorescence intensities compared with those from wells containing p53(C–C) and p53(C–^m^C). The ICON probe fixed on the well bottom distinguished 5′-^m^C from 3′-^m^C and detected only methylation of 5′-C regardless of methylation of 3′-C.

## 4. Conclusions

We have described a new, high-value aspect of on-chip methylation analysis through osmium-DNA complexation. An ICON probe fixed onto a microwell formed a crosslink with the target 5-methylcytosine and assisted the detection using PCR amplification. The crosslink was sequence-selective and completely independent of the other methylation site. Although there remain further aspects to be examined toward realizing an easier-to-use methylation analysis, such as optimization of PCR conditions suitable for ICON, this on-chip assay supported by the chemical basis could be an important component of the next generation of high-throughput methylation analyses.
